# Environmental Influence and Recruitment Bias in Studies on Internet Addiction. Comment on “Addiction Symptom Network of Young Internet Users: Network Analysis”

**DOI:** 10.2196/44438

**Published:** 2023-07-11

**Authors:** Ting Yun Huang, Yung-Po Liaw

**Affiliations:** 1 School of Medicine Chung Shan Medical University Taichung City Taiwan

**Keywords:** internet addiction, Internet Addiction Test, network analysis, adolescents

## Letter to the Editor

We read with great interest the article published in the *Journal of Medical Internet Research* titled, “Addiction Symptom Network of Young Internet Users: Network Analysis” by Lu et al [1]. With the increased integration of the internet and society, the importance of understanding the impact of internet usage on the quality of our daily lives has become ever more paramount. Lu et al [1] used the Internet Addiction Test to evaluate 4480 participants. They reported significant differences when performing a network comparison of individuals with and without internet addiction (IA). They identified links between multiple core symptoms of IA, including impact on school work, job performance, boredom, self-control, and fantasies on the web. While these findings have broad implications on the interactions between various IA symptoms and how IA develops, we would like to recommend including additional details that could better clarify the results.

The study stated that participants were recruited from 2 universities in Jiangsu, China, via advertisement. We do not know the exact environment of the 2 universities that the participants were recruited from. Environmental factors are known to play a great role in the development of IA [2]. While there is substantial variability between the backgrounds of individual participants, it would be important to note the general environment that the participants interact with (ie, ease of access to internet cafés and outdoor activities, level of urbanization, etc) [3]. It may also be interesting to request more background information on each participant, if eligible. This would provide more depth to the analysis.

The authors should clarify the process and describe the implications of using advertisements as the method of recruitment. Participants in studies are often motivated by different factors, which could result in biases [4]. It should be noted that different methods of advertisement, such as recruitment via social media, may also result in biases [5].

In conclusion, we believe addressing the above points can help clarify the interpretation of the results. Lu et al [1] touch upon an important issue in which the understanding of IA development and symptoms is becoming critical in modern culture.

## Abbreviations

IA: internet addiction
